# Interventions to reduce the impact of unemployment and economic hardship on mental health in the general population: a systematic review

**DOI:** 10.1017/S0033291716002944

**Published:** 2016-12-15

**Authors:** T. H. M. Moore, N. Kapur, K. Hawton, A. Richards, C. Metcalfe, D. Gunnell

**Affiliations:** 1 School of Social and Community Medicine, University of Bristol, Bristol, UK; 2 NIHR CLAHRC West, University Hospitals Bristol NHS Foundation Trust, Whitefriars, Lewins Mead, Bristol,UK; 3 Centre for Suicide Prevention, Division of Psychology and Mental Health, The University of Manchester, Manchester,UK; 4 Centre for Suicide Research, Department of Psychiatry, University of Oxford, Warneford Hospital, Headington, Oxford,UK

**Keywords:** Anxiety, austerity, debt, depression, financial hardship, intervention, mental health, recession, self-harm, suicide, systematic review, unemployment

## Abstract

**Background:**

Job loss, debt and financial difficulties are associated with increased risk of mental illness and suicide in the general population. Interventions targeting people in debt or unemployed might help reduce these effects.

**Method:**

We searched MEDLINE, Embase, The Cochrane Library, Web of Science, and PsycINFO (January 2016) for randomized controlled trials (RCTs) of interventions to reduce the effects of unemployment and debt on mental health in general population samples. We assessed papers for inclusion, extracted data and assessed risk of bias.

**Results:**

Eleven RCTs (*n* = 5303 participants) met the inclusion criteria. All recruited participants were unemployed. Five RCTs assessed ‘job-club’ interventions, two cognitive behaviour therapy (CBT) and a single RCT assessed each of emotional competency training, expressive writing, guided imagery and debt advice. All studies were at high risk of bias. ‘Job club’ interventions led to improvements in levels of depression up to 2 years post-intervention; effects were strongest among those at increased risk of depression (improvements of up to 0.2–0.3 s.d. in depression scores). There was mixed evidence for effectiveness of group CBT on symptoms of depression. An RCT of debt advice found no effect but had poor uptake. Single trials of three other interventions showed no evidence of benefit.

**Conclusions:**

‘Job-club’ interventions may be effective in reducing depressive symptoms in unemployed people, particularly those at high risk of depression. Evidence for CBT-type interventions is mixed; further trials are needed. However the studies are old and at high risk of bias. Future intervention studies should follow CONSORT guidelines and address issues of poor uptake.

## Introduction

Job loss, debt and financial difficulties are associated with an increased risk of mental illness, self-harm and suicide (Fitch *et al.* 2011; Haw *et al.* 2015). During periods of economic recession the numbers of people affected by these and other problems rise and levels of depression, self-harm and suicide increase (Stuckler *et al.* 2009; Katikireddi *et al.* 2012; Chang *et al.* 2013; Corcoran *et al.* 2015). Interventions to help mitigate the effect of job loss and debt on mental health are an important element of policy response to periods of recession. Ecological studies indicate that factors such as government spending on active labour market programmes and unemployment protection schemes may counter the effect of recession on suicide rates (Stuckler *et al.* 2009; Norström & Grönqvist, 2014) and austerity measures such as reassessment of individuals’ eligibility for benefit could have the opposite effect (Barr *et al.* 2015). However, there have been few evaluations of specific interventions targeted at individuals.

A number of policy documents (WHO, 2011; van Stolk *et al.* 2014) have summarized some of the limited randomized controlled trial (RCT) evidence of the effects on mental health of interventions for people who have lost their jobs, most notably studies of the JOBS programmes in the USA (Caplan *et al.* 1989; Vinokur *et al.* 1995*b*) and the Työhön job search programme in Finland (Vuori *et al.* 2002). Two reviews assessed the evidence on ‘job search’ interventions and included data from randomized and non-randomized studies (Audhoe *et al.* 2010; Liu *et al.* 2014). Both reported that job search interventions improved depression and employment (Audhoe *et al.* 2010; Liu *et al.* 2014). However, to our knowledge, no systematic reviews have assessed evidence of the effectiveness of the range of interventions, including job-search programmes, designed to ameliorate the impact of job loss, unemployment and economic hardship on mental health.

Our aim was to systematically review the evidence from randomized controlled trials of interventions given to the general population to reduce the effects of economic hardship on mental health. Our focus was on studies conducted in general population samples of working age individuals, rather than those focusing on specific high-risk samples, such as individuals with serious mental illness (Kukla & Bond, 2009; Tsang *et al.* 2010; Burke-Miller *et al.* 2012; Nieuwenhuijsen *et al.* 2014). We also excluded studies on select population groups (e.g. single mothers) where we felt policy responses and interventions would be tailored specifically for the particular needs of those populations and were not generalizable to a general population (Wiggins *et al.* 2004; Forgatch & DeGarmo, 2007). This review will be of use to policy makers, researchers planning future intervention studies and public health practitioners working in local authorities.

## Method

### Criteria for considering studies for this review

We included randomized controlled trials (RCTs) and cluster randomized trials of public health or health service interventions designed to mitigate the effects of unemployment, debt or austerity measures in the general population. We included only studies with a measure of mental health as an outcome, such as studies with measures of either mental disorder or mental health symptom scales. Examples of types of intervention include: group support or workshops to provide people with job search skills and resilience to the impact of rejected applications; advice type interventions (e.g. Citizens Advice Bureau) to help people navigate their way through benefits systems and/or access relevant support or to provide debt advice; interventions aimed at training frontline staff in job centres or benefits agencies or debt collection agencies to identify individuals who have mental health problems and help them respond appropriately. We excluded studies focused on people with serious mental illness, as this is a distinct subgroup of the population requiring specific intervention types; people not of working age; rehabilitation interventions for people with somatic or mental health problems that either aimed to help them get back into work, or to prevent them from losing their job if they were currently employed; and interventions aimed at selected specific groups of the working population (e.g. single mothers). We also excluded studies if the authors did not report any measure of mental health. A protocol for the review was registered in advance on the PROSPERO website, we prepared the review following Cochrane methods and using PRISMA reporting guidelines (Liberati *et al.* 2009; Higgins & Green, 2011; Moore *et al.* 2015).

### Search

We searched MEDLINE, PsycINFO, Embase on Ovid; the Cochrane Library including CENTRAL on Wiley Interscience; and Science and Social Science Citation Index, and Arts and Humanities Citation Index on Web of Science. All databases were searched from inception to 27 May 2015 and re-run on 16 January 2016. We excluded letters, editorials, and conference proceedings for which there were no full-text papers. We searched the reference lists of, and ran a citation search on, all included studies. We used a combination of MeSH terms and text words for mental health combined with terms for economic hardship, unemployment, job insecurity credit advice and financial worries. We used filters for selection of RCTs taken from the Cochrane Handbook (Lefebvre *et al.* 2011). We did not exclude studies based on language (see Supplementary Appendix 1 for full details of the searches).

### Eligibility, data collection and assessment of risk of bias

We screened the titles and abstracts and eligibility of full-text reports independently and in duplicate (D.G. and T.H.M.M.) using a form to check the criteria and discussing any discordant decisions until consensus was reached. All authors extracted data and assessed risk of bias, independently and in duplicate, recording these on a data extraction form (D.G., T.H.M.M., K.H., N.K., C.M.). Disagreements were discussed until consensus was reached, with recourse to a third reviewer if necessary. To investigate bias we used the Cochrane Risk-of-Bias tool (Higgins *et al.* 2011). Domains assessed included quality of the random sequence generation, concealment of allocation, description of drop-outs and withdrawals, blinding (of participants, research personnel and outcome assessment) and selective outcome reporting. (See Supplementary Appendices for details of data extracted, eligibility and risk of bias assessment.)

### Synthesis

We planned to examine the treatment effect direction and consistency by providing a systematic narrative, structured summary of the evidence (tables and descriptive text) from the studies based on type of intervention and participants. There were insufficient data reported in the studies to prepare a meta-analysis for ‘job-club’ type interventions; the remaining interventions were too heterogeneous in terms of interventions to attempt to pool data. We categorized type of interventions on a *post-hoc* basis as described in our protocol because we were unclear what range of interventions, setting and participants we would identify (Moore *et al.* 2015).

## Results

**Fig. 1.**
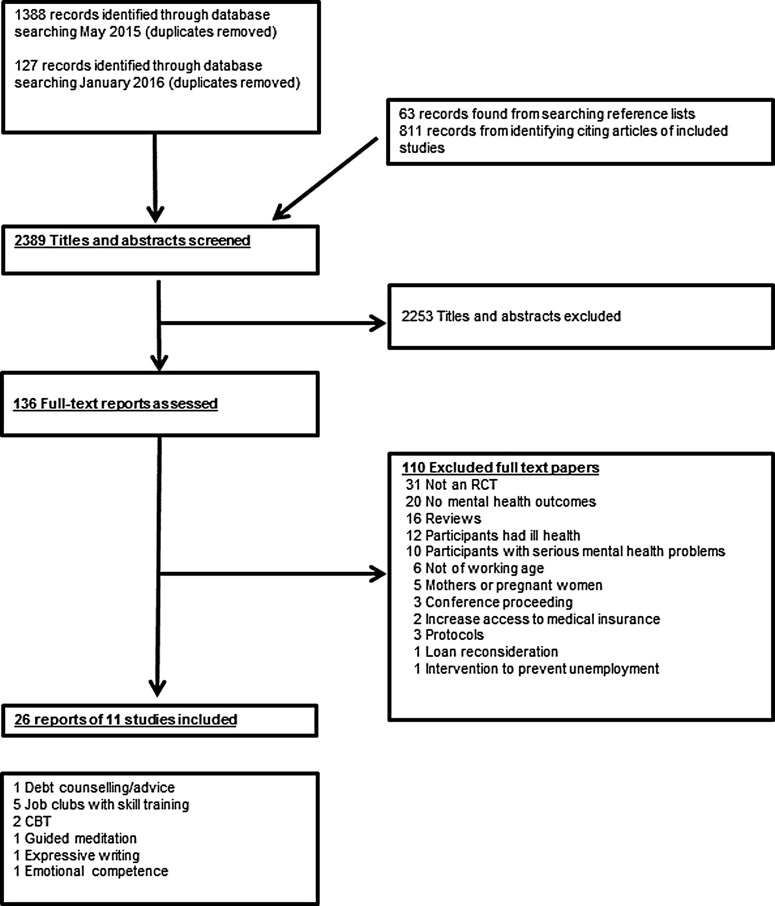
Study selection.

**Fig. 2.**
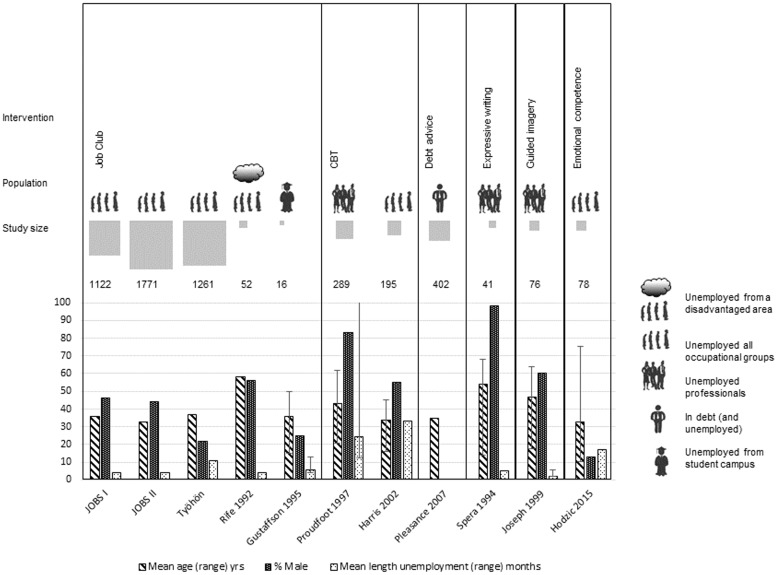
Visualization of study characteristics. Ranges for age and length of unemployment were presented when data were available.

Our search identified 2389 records (see Fig. 1). The 11 RCTs included 5303 participants and were reported in 26 papers (see Fig. 1). There was considerable heterogeneity in terms of type of intervention, and participants, study size (see Table 1, Fig. 2, Supplementary Appendix 2 and Supplementary Appendix 3). Six studies were from the USA, two from the UK, and one each from Spain, Australia and Finland. Five studies – four in the USA (Caplan *et al.* 1989; Rife, 1992; Gustafson, 1995; Vinokur *et al.* 1995*b*) and one in Finland (Vuori *et al.* 2002) – examined the effect of ‘job-club’ type interventions for unemployed people to cope with job loss and assisted them into new employment, two studies assessed the effects of cognitive behavioural therapy (CBT) for unemployed people (Proudfoot *et al.* 1997; Harris *et al.* 2002), one study investigated the effects of expressive writing (Spera *et al.* 1994), a second the effect of guided mental imagery (Joseph & Greenberg, 2001), and a third the effects of emotional competencies training (Hodzic *et al.* 2015*a*). Another study evaluated debt advice for people in debt (Pleasence & Balmer, 2007).

**Table 1.** Details of interventions and participants

Study ID, country, design, no. of arms, no. of participants	Population age and gender and ethnicity	Intervention type	Referral pathway and inclusion criteria	Control
JOBS I (Caplan *et al*. 1989; Vinokur *et al*. 1991*a*, *b*; Price *et al*. 1992; vanryn & Vinokur, 1992; Vinokur *et al*. 1995*a*, *b*) USA RCT 2 arms *N* = 1122 randomized, 83% completed baseline, 88% at 6 weeks and 80% at 4 months	Unemployed: 100% Duration of unemployment: mean of 13 weeks, s.d.=9 weeks. Maximum 4 months Age: mean 35.9 years (range not provided) Gender: 46% male Ethnicity: 75% white Baseline mental health: not reported	‘Job club’ (JOBS I) JOBS skills training seminars to improve job seeking Class based group active training sessions with two trainers (male/female pairs) aiming to cover problem solving, decision making processes, inoculation against setbacks, provision of social support and positive regard from trainers, learning and practicing job, search skills, building self-esteem. This intervention aimed to set itself apart from the work of Azrin et al (Azrin *et al.* 1975; Azrin & Philip, 1979) Delivered to: people in groups of 16–20 participants Duration and frequency: eight 3 h sessions over 2 weeks	From four state recruitment compensation offices in SW Michigan	Written material. 25 page booklet on Job skills and job searching
JOBS II (Vinokur *et al*. 1995*a*, *b*; Vinokur *et al*. 1996; Vinokur & Schul, 1997; Vinokur *et al*. 2000) USA RCT 2 arms *N* = 1771 completed baseline. 1801 randomized, 80% at T2; 87% at T3	Unemployed: 100% Duration of unemployment: mean 4.11 weeks s.d.=3.8 weeks. Maximum 13 weeks Age: mean 36.2 years (range not provided) Gender: 45% male Ethnicity: 76% white Baseline mental health: People with depression were excluded. Each participant completed a selection questionnaire from Price *et al*. (1992) (JOBS I) and a ‘weighting’ was applied to classify people at low or high risk of poor mental health. JOBS II then oversampled from those at High risk of poor mental health based on these criteria. This was done because JOBS I found the Job Club intervention appeared to improve depression symptoms of those at higher risk	‘Job club’ (JOBS II) JOBS skills training seminars to improve job seeking Content same as JOBS I but with a focus on increasing sense of mastery, increase of personal control and job search efficacy. Included problem solving, decision making, group processes, inoculation against setbacks, receiving social support and positive regard from the trainers. Learning and practicing job seeking skills. Skill training Group support, problem solving, trainer support, active identification of possible setbacks, planning how to respond to these and practicing responses Delivered to: People in groups of 12–20 participants Duration and frequency: 5x4 h sessions over 1 week	From four state recruitment compensation offices in South West Michigan	Written, self-administered, job search materials and pamphlet. 25 page booklet on Job skills and job searching
Työhön (Vuori *et al*. 2002; Vuori & Vinokur, 2005) Finland RCT 2 arms *N* = 1261 randomized, 994 (78%) completed	Unemployed: 100% Duration of unemployment: median 5 months (mean = 10.7 s.d. = 17.3). 28% unemployed for 12 months or longer Age: mean = 37 (18–61 years) Gender: 22% male Ethnicity: not reported Baseline mental health: not reported	‘Job club’: Työhön ‘let's get to work’ Job Search Program. Based on JOBS I Main difference between this trial and JOBS I and JOBS II is context. Finnish unemployed participants have longer access to higher rates of unemployment benefit than US counterparts Components: class based group active training sessions with two trainers (male/female pairs) aiming to cover problem solving, decision making processes, inoculation against setbacks, provision of social support and positive regard from trainers, learning and practicing job, search skills, building self-esteem Delivered to: groups of 6–17 participants Duration and frequency: daily, half-day sessions over 5 days	By post, phone and direct contact from four employment offices by contacting recently laid-off workers; recruiting services of trade unions, associations of the unemployed, and universities; and by advertisements in newspapers, radio, and the Internet	Written job search materials
(Rife, 1992; Rife & Belcher, 1994) USA RCT 2 arms *N* = 52, randomized 100% completed	Unemployed: 100%. Duration of unemployment not described Age: mean 58 years (range not provided) Gender: 56% male Ethnicity: 94% white Baseline mental health: mean Geriatric Depression Scale (GDS) intervention 6.6 control 6.2. Participants were reported to be ‘mildly depressed’	‘Job club’: JOBS skills training seminars to improve job seeking Provision of practical assistance in completing forms and CVs and access to telephones to call potential employers· Based on work by Gray 1983 and Azrin 1978(Azrin, 1978, Gray, 1983) Components: ‘Job club’ consisted of a half-day, group, workshop on job-search techniques. Then on-going ‘job club’ meetings two afternoons per week. These comprised goal setting, receiving information about job seeking, interviewing, writing CVs and completing application forms. Groups also provided practical advice and help on information on job leads, access to telephones, and support (peer and from the group leaders) Duration and frequency: one half day workshop followed by two afternoon workshops per week for 12 weeks	People who had applied for employment assistance services with the community agency	Usual service: State Government Job Service and community referral programme, e.g. employment registration, information and referral
(Gustafson, 1995) USA RCT 2 arms *N* = 16 100% completed	Unemployed: 100% Duration of unemployment: mean 5.5 months range (1.5–13) Age: median 36 years (21–50 years) Gender: 25% male Ethnicity: 88% white Baseline mental health Stait Anxiety: Intervention mean 48.2 (s.d.=11.8), control mean 41.0 (s.d.=8.5). Trait anxiety mean 43.6 (s.d.=14.3), control mean 46.6 (s.d.=7.5)	‘Job club’: based on JOBS I Components: coping skills, problem solving, inoculation against setbacks, social support and positive regard from trainers, job seeking skills training and practice, job interview preparation Delivered to: group of 8 people Duration and frequency: 8x3 h sessions over 2 weeks	Recruitment from Saddleback college career centre offices, California	Written, self-administered, job search materials and pamphlet
(Proudfoot *et al*. 1997, 1999) UK RCT 2 arms *N* = 289 randomized	Unemployed: 100% Duration of unemployment: for 12 months to 12 years. Mean length of unemployment 25.8 months intervention group and, 23.1 months control group Age: mean 43 years (22–62 years) Gender: 83% male Ethnicity: not reported Baseline mental health: Intervention group: GHQ-30 59% scored 5 or more. Control group: GHQ-30 54% scored of 5 or more (psychiatric caseness)	CBT Group CBT To identify and test the validity of automatic thoughts, reattribution of thoughts, and monitoring behaviour and experimentation with behaviours. The CBT included weekly homework Components: introduction to cognitive model, goal setting, automatic thoughts, Though recording, common thinking errors, techniques to change unhelpful thinking, personalization of the approach, homework, Final session was to teach participants to use what they had learned in the future Delivered to: groups of 10–15 people Duration and frequency: 3 h weekly for 7 weeks	Newspaper adverts, mail shots, the UK Employment service, employment/recruitment organization	Social support programme, same format as CBT and included weekly homework
(Harris *et al*. 2002) Australia RCT 2 arms *N* = 195 randomized 100 % completed	Unemployed: 100% Duration of unemployment: mean length of unemployment 33 months (s.d. = 40.0) Age: 34 years s.d. = 9.4 (18–45 years) Gender: 55% male Ethnicity: not reported Baseline mental health Intervention SF-36 Mental Component Scale 41.03 (15.13), control 45.63 (12.35) Other: long-term unemployed from disadvantaged areas of Sydney. 51% left school before completing higher-level qualifications	CBT: Group CBT Cognitive restructuring (identifying negative thoughts, modifying and replacing thoughts), problem solving (five step structured problem solving activity), behaviour strategies (relaxation skills, breathing techniques) CBT was modified during pilot testing from an existing 3-day CBT programme to a 2 day programme Delivered to: groups of 5–16 people Duration and frequency: 2 days (11 h total)	Employment support agencies in disadvantaged areas of Sydney	Two-day Senior First Aid certificate. Two-day Senior First Aid Certificate – fundamental principles/knowledge/skills of First Aid Duration and frequency: Two days with exam
(Pleasence & Balmer, 2007) UK RCT 2 arms *N* = 402 randomized. 234 completed (119 intervention and 115 control)	Unemployed: ‘Mostly unemployed seeking work’ recruited from job centres In debt: 100% of participants were in debt Age: mean 35 years (range not provided) Gender: not reported Ethnicity: 66% white Baseline mental health: not reported	Debt advice: telephone call from trained advisors from ‘National Debtline’ Where was advice given? By phone Content: telephone call from National Debtline. Advice was free of charge. Immediate advice and assistance was provided in relation to any emergency issues, (e.g. bailiffs or repossession). Main advice was on longer-term resolution of problems such as debt management programmes. Written self-help materials were provided. Participants could be referred on to other services Delivered to: individuals Duration and frequency: one-off telephone call	Researchers approached people in job centres	No intervention. Usual Job Centre service
(Spera *et al.* 1994)USA RCT 3arms^a^ *N* = 63 (in three arm, 41 participants discounting the non-randomized intervention arm)	Unemployed: 100% Duration of unemployment: mean 5 months. Mean 20 years working with same employer Age: mean 54 years (40–68 years) Gender: 98% male Ethnicity: not reported Baseline mental health: measured but values not reported Other: managerial professionals being offered the services of an outplacement company as they were being laid off from employment	Writing transition project Daily, private, disclosive writing sessions for recording personal and deepest thoughts and feelings about unemployment and how their lives both personal and professional, had been affected. Participants were encouraged to explore their emotions deeply Duration and frequency: 20 min daily for 5 days	Professionals recruited from outplacement firms	Control writing: Daily, private, disclosive writing sessions for recording plans for the day, and activities in job search Duration and frequency: 20 min daily for 5 days
(Joseph, 1999; Joseph & Greenberg, 2001) USA RCT 2 arms *N* = 76 randomized.	Unemployed: 100% Duration of unemployment: mean unemployment duration 2.3 months, range (0–5.8 months) Age: mean 46.8 years (29–64 years) Gender: 60% male Ethnicity: 83% white Baseline mental health: Intervention group mean CES-D (s.d.) 12.38 (9.39) Control CES-D 16.68 (10.25) Other: participants were described as ‘business workers’. Mean years in last job 8.27 years (0.17–30.58) years	Guided imagery group intervention: Components: six brief, 20 min sessions. Participants followed tape recorded guided muscle relaxation followed by guided imagery covering; emotional experience of job loss and unemployment, job search and job interview rehearsal, positive self-regard and cognitive reframing	Professionals recruited from outplacement firms	Placebo imagery: Self-directed visualization of job search plans and activities in the past and the future 6x20 min ‘imagery sessions’ following written instructions, thinking about their daily job search activities
(Hodzic *et al*. 2015*a*, *b*) Spain RCT 2 arms *N* = 78, randomized	Unemployed: 100% Duration of unemployment: mean unemployment duration 16.9 months. Age: intervention mean age 32.68 (s.d.=10.34), control mean age 36.4 (s.d.=12.02) Gender: 13% male Ethnicity: not reported Baseline mental health: GHQ-12 intervention group mean = 2.42 (s.d. = 0.53), control group mean = 2.58 (s.d. = 0.60). Mood anxiety mean = 2.94 (s.d. = 0.98), control mean = 2.59 (s.d. = 0.95) Mood depression mean = 1.83 (s.d. = 0.87), control mean = 1.70 (s.d. = 0.58)	Emotional competences training: based on Kotsou *et al*. (2011) Group work to identify and understand emotions of the self and other, using emotions to assess need, regulating emotions, conflict management, practical work to regulate one's own emotions and to listen to others’ emotions. Including video clips, group exercise, discussion and role play Duration and frequency: 3 days (5 h per day). Day one and 2 were consecutive. Two weeks gap between day 2 and day 3	Unemployed from unemployment agencies Inclusion criteria. (a) being unemployed, (b) motivated about the intervention process, (c) no prior knowledge about the intervention, (d) not using the intervention content for professional purposes, (e) not planning psychological treatment during the training, and (f) not being dependent on drugs, alcohol or psycho-pharmaceuticals	No intervention

a Although Spera *et al*. (1994) had three arm study with *n* = 63 participants we are using data from just two study arms (*n* = 41) the people in one of the groups (no writing) were not allocated during the randomization procedure (Spera *et al.* 1994). CES-D Centre of epidemiologic studies depression scale.

We excluded 110 reports (see Fig. 1 and Supplementary Appendix 4). Twenty papers describing RCTs were excluded because they did not report any mental health data (see Supplementary Appendix 4); five reports because the population, i.e. single or new mothers on benefits, would necessarily have interventions tailored to suit their circumstances (Wiggins *et al.* 2004; Forgatch & DeGarmo, 2007; Morris & Hendra, 2009; Jagannathan *et al.* 2010; Kneipp *et al*. 2011); one report providing loans (Fernald *et al.* 2008); and two reports where the intervention was increasing access to healthcare insurance (Finkelstein *et al.* 2012; Baicker *et al.* 2013), as these interventions were unlikely to be suitable for a general population.

Despite searching for and including people in the general population all participants in the included studies were unemployed, with mean durations of unemployment ranging from 2.3 to 33 months (see Fig. 2). Three studies recruited professionals or management-level staff (Spera *et al.* 1994; Proudfoot *et al.* 1997; Pleasence & Balmer, 2007). Mean age ranged from 32 (Vinokur *et al.* 1995*b*) to 58 (Rife, 1992) years and gender balance varied from 13% male (Hodzic *et al.* 2015*a*) to 98% male (Spera *et al.* 1994).

**Fig. 3.**
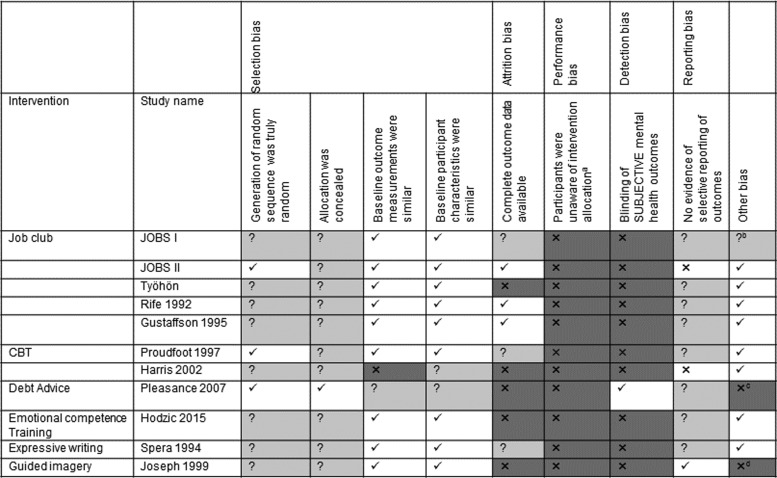
Risk of bias. ✓ Domain was judged to be low risk of bias; ×, domain was judged to be at high risk of bias; ?, it was not possible to assess the risk of bias for this domain; ^a^it is not possible to obscure the type of intervention in studies such as these as the participants are aware of the intervention they are receiving, therefore all studies are rated at high risk-of-bias; ^b^employment outcome was complex, dichotomised to working enough and not working enough however 14% of people did not meet these criteria and were not included in the outcome assessment; ^c^stopped early high drop out; ^d^difference in baseline of people exposed to relaxation techniques. CBT, Cognitive behavioural therapy.

Most studies were assessed as at high or unclear ‘risk of bias’ so the numerical outcomes need to be interpreted with some caution. Information needed to assess bias was not reported in several studies and participants in all 11 studies would have been aware of the intervention they were given and so all the studies were at high risk of bias for that domain (see Fig. 3).

Ten of 11 studies described the personnel delivering the intervention the exception was Rife (1992), while just two, JOBS II and Työhön, described their training (Vinokur *et al.* 1995*b*; Vuori *et al.* 2002). Two studies, JOBS I and JOBS II reported supervision for their staff (Vinokur *et al.* 1995*a*, *b*). Four studies, JOBS I, JOBS II, Työhön and Harris *et al*. (2002) reported use of a manual (Caplan *et al.* 1989; Vinokur *et al.* 1995*b*; Harris *et al.* 2002; Vuori *et al.* 2002) and three (JOBS I, JOBS II and Työhön) included an assessment of fidelity to treatment (Caplan *et al.* 1989; Vinokur *et al.* 1995*b*; Vuori *et al.* 2002) (see Supplementary Appendix 5).

### ‘Job-club’ interventions

Five studies, reported in 17 papers, assessed the effects of ‘job-club’ interventions. Four of these compared ‘job-club’ to written, self-administered job-search materials (JOBS I, JOBS II, Työhön and Gustafson, 1995) and one, Rife *et al.* compared ‘job club’ to usual unemployment centre services. The JOBS I intervention (Caplan *et al.* 1989; Vinokur *et al.* 1991*a*, *b*, *1995a*; Price *et al.* 1992; Vanryn & Vinokur, 1992) (*n* = 1122) delivered job skills training seminars to groups of 16–20 people in eight sessions of 3 h (Table 1, Fig. 2 and Supplementary Appendix 3). The JOBS I intervention was modified in JOBS II (*n* = 1771) to focus more on enhancement of personal control, sense of mastery and job-search self-efficacy; sessions were reduced from eight sessions over 2 weeks in JOBS I to daily 4 h sessions provided over 5 days in JOBS II and training of group facilitators was also increased (Vinokur *et al.* 1995*b*, 1996, 2000; Vinokur & Schul, 1997). Both JOBS I and JOBS II excluded people with any signs of mental illness (JOBS I; Caplan *et al.* 1989) or depression scores of >3 on Hopkins Symptom checklist 90, (JOBS II; Vinokur *et al.* 1995*b*) at baseline. Vuori *et al*. (2002) adapted the JOBS II intervention for use in Finland (*n* = 1261), named it Työhön (‘let's get to work’), and recruited people with a longer history of unemployment (11 months *v.* 3-4 months in JOBS I and JOBS II). Unlike the two JOBS trials a high proportion (78%) of participants were female (Vuori *et al.* 2002; Vuori & Silvonen, 2005; Vuori & Vinokur, 2005). Rife *et al.* (*n* = 52) provided practical job skills training workshops two afternoons per week for 12 weeks (Rife, 1992; Rife & Belcher, 1994) and Gustafson *et al.* (*n* = 16) delivered job skills training interventions similar to JOBS I for eight 3 h sessions over 2 weeks (Gustafson, 1995).

The ‘job club’ intervention delivered in JOBS I had no effect on levels of anxiety or depression at 6 weeks or 4 months (see Table 2). A *post-hoc* subgroup analysis showed that the participants with higher risk of developing depression, based on the 25% of participants with highest baseline risk of depression, economic hardship and social assertiveness (Price *et al.* 1992) benefited most from the intervention (interaction: *p* = 0.01). In high-risk participants depression scores were reduced at 6 weeks [difference in means: −0.26, 95% confidence interval (CI) −0.48 to −0.04; interaction: *F* = 6.07 *p* = 0.01], 4 months (difference in means: −0.36, 95% CI −0.59 to −0.13; interaction: *F* = 12.14 *p* = 0.001) and 28 months (difference in means: −0.25, 95% CI −0.50 to 0.0; interaction: *F* = 6.05 *p* = 0.01) by up to almost 0.5 s.d. on the depression subscale of the Hopkins Symptom Checklist 90 (HSCL-90; see Table 2). JOBS I had no effect on employment at the same time points (see Table 3) (Caplan *et al.* 1989; Price *et al.* 1992; Vinokur *et al.* 1991*a*, *b*, *1*995*a*; Vanryn & Vinokur, 1992).

**Table 2.** Mental health outcomes by Intervention type

						Intervention		Control			Calculation of difference in means, or risk difference, using available published summary data from studies^a^^,^^b^
Intervention type Outcome		Outcome scale	Study	*n*	Time point (sample)	Mean (s.d.)	*n*	Mean (s.d.)	*n*	Published analysis by trial authors	
Job club	Anxiety	Subscale of HSCL-90	JOBS I	630	6 weeks (all)	1.87 (–)	–	1.88 (–)	–	ES = −0.03, *t* = 0.36(630df), *p* = 0.718	MD −0.01 (not calculable)
				623	4 months (all)	1.89 (–)	–	1.86 (–)	–	ES = 0.04, *t* = 0.45(623df), *p* = 0.652	MD −0.03 (not calculable)
		STAI State	Gustafson 1995	16	6 weeks	43.7 (14.9)	8	38.0 (6.9)	8	χ^2^ *p* > 0.05	MD 5.70 (−5.68 to 17.08)
		STAI Trait		16	6 weeks	41.7 (10.7)	8	44.6 (7.3)	8	χ^2^ *p* > 0.05	MD −2.90 (−11.88 to 6.08)
	Depression	HSCL-90	JOBS I	630	6 weeks (all)	1.84 (–)	–	1.91 (–)	–	ES = −0.09, *t* = −1.12(630df), *p* = 0.263 ns	MD −0.07 (not estimable)
				522	6 weeks (low)^c^	1.67 (0.50)	340	1.60 (0.51)	182	Interaction *F* = 6.07, *p* = 0.01^d^	MD 0.07 (−0.02 to 0.16)
				179	6 weeks (high)^c^	2.21 (0.73)	117	2.47 (0.70)	62		MD −0.26 (−0.48 to −0.04)
				623	4 months (all)	1.84 (–)	–	1.92 (–)	–	ES = −0.11, *t* = −1.30(623df), *p* = 0.194 ns	MD −0.08 (not estimable)
				695	4 months (all)	1.72 (0.63)	465	1.84 (0.69)	230	*t* test ns	MD −0.13 (−0.24 to −0.02)
				511	4 months (low)^c^	1.59 (0.55)	343	1.63 (0.52)	168	Interaction *F* = 12.14, *p* = 0.001^d^	MD −0.04 (−0.14 to 0.06)
				184	4 months (high)^c^	2.08 (0.72)	122	2.44 (0.78)	62		MD −0.36 (−0.59 to −0.13)
				456	28 months (low)^c^	1.55 (0.53)	305	1.61 (0.56)	151	Interaction *F* = 6.05, *p* = 0.01^d^	MD −0.06 (−0.17 to 0.05)
				157	28 months (high)^c^	1.95 (0.73)	103	2.20 (0.77)	52		MD −0.25 (−0.50 to 0.00)
		HSCL-90	JOBS II	742	2–6 months (low)	–	–	–	–	–	–
				470	2–6 months (high)	–	–	–	–	*F*_1,1331_ = 4.10 *p* = 0.043, difference = −0.2 s.d. at 2 months Difference = −0.22 s.d. at 6 months^e^ -	–
				578	24 months (all)	–	–	–	–	Beta LR = −0.06, *p* < 0.05	–
		CIDI MDE^f^		578	24 months (all)	–	–	–	–	Beta LR = −0.04, *p* > 0.05	–
		CIDI Probable MDE^f^		578	24 months (all)	–	–	–	–	Beta Log R = −0.49, *p* < 0.05 (OR = 0.612)	–
		GDS	Rife 1992	52	12 weeks	5.03 (–)	26	7.07 (–)	26	MW U 188.5, *Z* = −2.76, *p* < 0.05	MD −2.0 (not estimable)
		HSCL90	Työhön	1049	6 months	–	–	–	–	SLRC (Beta) = −0.04 ns	
				1261	24 months	–	–	–	–	SLRC (Beta) = −0.06, *p* < 0.05	
	Psychological symptoms	GHQ-12	Työhön	1049	6 months	–	–	–	–	SLRC (Beta) = −0.06, *p* < 0.05	
				952	24 months	–	–	–	–	SLRC (Beta) = −0.06, *p* < 0.05	
CBT	Psychiatric caseness	Score >5 on GHQ-30	Proudfoot 1997	209	7 weeks	–	–	–	–	21% CBT 23% control *t* test, *p* = 0.78	RD−0.01 (−0.12 to 0.09)
	Psychiatric symptoms	GHQ-30		209	7 weeks	3.72 (5.81)	112	5.16 (7.01)	97	*F* = 3.91, *p* < 0.05^g^	MD −1.44 (−3.20 to 0.32)
				183	3 months	4.92 (7.21)	94	6.58 (8.14)	89	*F* = 3.85, *p* < 0.05^g^	MD −1.66 (−3.89 to 0.57)
		SF-36 MCS	Harris 2002	195	3–4 months	44.14 (12.19)	57	46.31 (12.78)	43	*t* test ns^h^	MD −2.17 (−7.13 to 2.79)
	Mood	BHS	Harris 2002		3–4 months	5.11 (4.27)	57	3.07 (2.73)	43	*F*_1, 97_ = 7.26, *p* = 0.01^g^	MD 2.04 (0.66 to 3.42)
		LSS Opt			3–4 months	13.54 (4.09)	57	16.14 (3.54)	43	*F*_1, 97_ _=_ 7.29, *p* = 0.01^g^	MD −2.60 (−4.10 to −1.10)
Debt advice	Anxiety	STAI-6	Pleasence 2007	402^i^	0–5 months	–	–	–	–	MVM ES -2.43, s.e. = 2.73 ns	
Emotional competence training											
	Mental health	GHQ-12	Hodzic 2015	75	1 months	2.18 (0.59)	41	2.48 (0.60)	34		MD −0.30 (−0.57 to 0.03)
					6 months	2.20 (0.53)	38	2.38 (0.53)	26		MD 0.18 (−0.44 to 0.08)
	Stress	PSSS		75	1 months	2.73 (0.66)	41	2.69 (0.66)	34		MD 0.04 (−0.26 to 0.34)
				64	6 months	2.61 (0.60)	38	2.69 (0.56)	26		MD −0.08 (−0.37 to 0.21)
	Anxiety	POMS		75	1 months	2.63 (0.92)	41	2.73 (0.98)	34		MD −0.10 (0.53 to 0.33)
				64	6 months	2.64 (1.02)	38	2.76 (0.86)	26		MD −0.12 (−0.58 to 0.34)
	Depression	POMS		75	1 months	1.76 (0.75)	41	1.76 (0.85)	34		MD 0.00 (−0.37 to 0.37)
				64	6 months	1.72 (0.81)	38	1.67 (0.55)	26		MD 0.05 (−0.28 to 0.38)
Guided imagery	Depression	CES-D	Joseph 2001	52	2 weeks	10.67 (10.63)	26	12.92 (11.02)	26	MF ANOVA no main effect intervention *F*_1,47_ = 2.25, *p* = >0.05 No intervention x time interaction *F*_2,94_ = 0.062, *p* = 0.939 ns	MD −2.25 (not estimable)
				52	2 months	8.71 (8.86)	26	12.48 (12.26)	26	ANOVA result as above	MD −3.77 (not estimable)
Expressive writing	Anxiety	SMUHQ	Spera 1994^i^	41^j^	3 and 8 month	–	–	–	–	ANOVA ns	–

BHS, Beck Hopelessness Scale; CES-D, Centre of Epidemiologic Studies Depression Scale; ES, Effect size; GDS, Geriatric Depression Scale; GHQ-12, General Health Questionnaire - 12 items. Self-report measure of psychological morbidity (Goldberg *et al.* 1997); GHQ-30, General Health Questionnaire 30 Item. Self-report measure of psychological morbidity; HSCL-90, Hopkins Symptom Checklist 90 Subscale – 11 items adapted for use in Finland (Vuori *et al.* 2002). The measure of depressive symptoms was a 10-item Finnish scale (Salokangas *et al.* 1994) based on the Hopkins Checklist (Derogatis *et al.* 1974) Cronbach's α coefficients were 0.92 at T1 and 0.92 at T4; Log R, Logistic regression; LR, Linear Regressions; LSS-Opt, Life satisfaction scale subscale optimism; MD, difference in means; MF ANOVA, multifactorial ANOVA; MVM, multivariate model, Pleasence & Balmer (2007). The authors state ‘We fitted a multivariate model fitting STAI-6 and EQ-5D scores simultaneously as normal response variables’ The effect sizes they report are changes from baseline to follow-up at 20 weeks; MW-U, Mann–Whitney *U*; ns, not reaching the statistical significance of a *p* value ⩽0.05; OR, odds ratio; POM, Profile of Mood States Questionnaire; PSS, Perceived Stress Scale; RD, risk difference; SF-36, Short-Form-36 Health Survey Questionnaire (Ware *et al.* 1994); SLRC, Standardized linear regression coefficient; For the Työhön study the s.d. for the HSCL-90 were reported as 6 for the intervention or 6.5 for the control. Therefore a change in 0.06 of a s.d. represents a change in score of 0.36 of a point on the HSCL-90 scale). For the JOBS II study no s.d. is provided for the HSCL-90; SMUHQ, Southern Methodists University Health Questionnaire (Watson & Pennebaker, 1989); STAI, Stait Trait Anxiety Scale (reduction in score = benefit range 20–80 score of >42 = case). Gustafson 1995; 6 week data (Gustafson, 1995): Harris 2002; 3–4 month data (Harris *et al.* 2002): Hodzic 2015; 1 month and 6 month data (Hodzic *et al*. 2015*a*): JOBS I; 6 week, 4 month data (Vinokur *et al*. 1991*a*), 28 month data (Vinokur *et al*. 1991*b*), low risk and high risk data all time points (Price *et al*. 1992): JOBS II; 2 month and 6 month data (Vinokur *et al.* 1995*b*), 2 year data (Vinokur *et al*. 2000): Joseph 2001; 2 week and 2 month data (Joseph, 1999, Joseph & Greenberg, 2001): Rife 1992; 12 week data (Rife, 1992): Työhön; 6 month data (Vuori *et al*. 2002); 24 month data (Vuori & Silvonen, 2005): Pleasance 2007; 0-5 month (Pleasence & Balmer, 2007): Proudfoot 1997; 7 week data (Proudfoot *et al*. 1999, Proudfoot *et al.* 1997): Spera 1994 (Spera *et al*. 1994).

a Risk difference calculated using methods described in Deeks & Higgins (2010) Statistical algorithms in Review Manager 5.2 (Deeks & Higgins, 2010).

b Mean difference calculated using methods described in Salanti (2013) Statistical algorithms for the Calculator in Review Manager 5.2 (Salanti, 2013).

c Data from Price *et al.* (1992). Participants were at high or low risk of depression. NB People scoring ⩾3 on pre-test depression were excluded from the analysis.

d Two-way ANCOVA (analysis of covariance) baseline depression and hours of employment as covariates and stratified by predicted risk of depression score (75% low risk; 25% high risk) (Price *et al.* 1992).

e Generalized linear model analysis of variance (ANOVA) 4.10 = *F* of interaction of risk (high or low) and condition (intervention or control) (Vinokur *et al.* 1995*b*).

f CIDI (Composite Index of Depression Inventory). Defines the occurrence of major depressive episode (MDE). A less stringent definition of ‘probable’ MDE was defined by dichotomizing a score of 0–6 = 0 or no diagnosis whilst 7–8 = 1 or probable diagnosis MDE.

g ANCOVA (analysis of covariance) with pre-test scores as covariates.

h Harris *et al.* do not present the *p* values for the *t* tests for SF-36 MCS. The *t* test is for baseline to follow-up within groups, i.e. not comparing groups. The authors report a χ^2^ analysis to test for differences between groups but these data were not presented. They also prepare an ANCOVA using baseline values as covariates – and do not present these data.

i Only 31% of the intervention group received debt advice. 10% of the control group sought and obtained debt advice.

j Although the Spera *et al.* (1994) study had three arms (*n* = 63) only two of the arms (Writing and No writing *n* = 41) were allocated at random. While we are reporting only from these two study arms the ANOVA analyses include all three arms.

**Table 3.** Employment, debt and debt awareness outcomes by intervention type

Intervention type	Study	Outcome	Time point	*n*	Intervention %	Control %	Statistical evidence for differences	Calculation of difference in means, or risk difference, using available published summary data from studies^a^^,^^b^
Job club	JOBS I	Employment^c^	6 weeks	563	33	26	*t* test ns^d^	RD 0.07 (0.00–0.14)
			4 months	499	60	51	*t* test ns^d^	RD 0.08 (−0.00 to 0.16)
			28 months		71.2	68.3	MANOVA *F* = 1.25 ns^e^	Not estimable
	Gustafson 1995	Employment	6 weeks	16	63	50	χ^2^ *p* > 0.05	RD −0.13 (−0.61 to 0.36)
	JOBS II	Employment^c^	2 months		34	27	Wald's χ^2^ = 4.44, *p* > 0.05	RD 0.07 (not estimable)
			2 months		35	29	Wald's χ^2^ = 5.79, *p* > 0.05	RD 0.06 (not estimable)
			6 months		63	67	Wald's χ^2^ = 4.13, *p* < 0.05	RD 0.04 (not estimable)
			6 months		62	54	Wald's χ^2^ = 4.55, *p* < 0.05	RD 0.08 (not estimable)
			24 months	–	–	–	BLRC = 0.44, OR = 1.55 *p* < 0.001	Not estimable
	Rife 1992	Employment	12 weeks	52	65	26	χ^2^ (1) = 7.73, *p* < 0.01	RD 0.38 (0.13–0.63)
	Työhön	Employment	6 months	1261	34	31.9	ns	RD 0.02 (−0.04–0.08)
			24 months	1112	54.1	49.5	χ^2^ = 2.41 ns	RD 0.05 (−0.02 to 0.11)
CBT	Proudfoot 1997	Employment	4 months	209	34	13	χ^2^ *p* = 0.0006	RD 0.21 (0.09 to 0.32)
		FT PT temp	4 months	209	49	28	χ^2^ *p* = 0.0016	RD 0.21 (0.08–0.34)
	Harris 2002	Employment activity^f^	3–4 months	100	–	–	χ^2^ = 0.27, *p* = 0.35	Not estimable
Guided imagery	Joseph 2001	Employment	2 months	52	62	11	χ^2^ = 14.02, *p* < 0.001	RD 0.50 (0.28–0.72)
			4 months	124	69	38	*X*^2^ = 5.79, *p* < 0.001	RD 0.31 (0.05–0.57)
Expressive writing	Spera 1994^g^	Employment	3 months	41^g^	25	0	*t*_50_ _=_ 2.10, *p* = 0.04; ANOVA *F*_2,59_ _=_ 3.72, *p* = 0.03^f^	RD 0.25 (0.05–0.45)
			8 months	41^g^	52.6	23.8	–	RD 0.29 (−0.00 to 0.58)
		FT; PT and contract	8 months	41^g^	68.4	47.6	ANOVA *F*_2,59_ _=_ 3.72, *p* = 0.03^h^	RD 0.22 (−0.07 to 0.52)
Emotional competence training	Hodzic 2015	Employment	6 months	63	21.2% (7)	10% (3)	χ^2^ = 1.48, *p* = 0.22	RD 0.11 (−0.06 to 0.29)
			12 months	63	36.4% (12)	10% (3)	χ^2^= 6.02, *p* = 0.01	RD 0.26 (0.07–0.46)
Debt advice	Pleasance 2007	Facing a debt problem	0–5 months	234	35	37	χ^2^2 = 0.22, *p* = 0.64	RD 0.92 (0.65–1.30)
		Perceived changes in financial circumstances ‘better’	5 months	234	42	30.1	ORM estimate intervention = 0.23, s.e. = 0.24, *p* = 0.34^i^	RD 0.12 (not estimable)
		Perceived changes in financial circumstances ‘the same’	5 months	234	17.6	24.8	χ^2^ = 5.92, *p* = 0.015	RD 0.07 (not estimable)
		See a future without debt	5 months	234	–	–	BLRA estimate intervention = 0.32, s.e. = 0.33 ns	
		Knowledge of debt problems	5 months	234	–	–	NRM intervention = 0.56, s.e. = 0.30, Wald test χ^2^ = 3.47, *p* = 0.06	
					Intervention mean	Control mean		
		Number of debts	5 months	234	2.29	2.16	Poisson model −0.03, s.e. = 0.10 ns	MD 0.13 (not estimable)

BLRA, Binary logistic regression analysis; BLRC, binary logistic regression coefficient; Contract, contract employment; FT, full time; NRM, normal response model; MD, difference in means; OR, odds ratio; PT, part time; RD, risk difference; Temp, temporary. Gustafson 1995; 6 week data (Gustafson, 1995): Harris 2002; 3 to 4 month data (Harris *et al.* 2002); Hodzic 2015; 6 month and 1 year data (Hodzic *et al*. 2015*b*): JOBS I; 6 week, 4 month data (Vinokur *et al*. 1991*a*), 28 month data (Vinokur *et al*. 1991*b*): JOBS II 2 month and 6 month data (Vinokur *et al.* 1995*b*); 2 year data (Vinokur *et al*. 2000): Joseph 2001; 2 week and 2 month data (Joseph, 1999; Joseph & Greenberg, 2001): Rife 1992; 12 week data (Rife, 1992): Työhön 6 month data (Vuori *et al*. 2002); 24 month data (Vuori & Silvonen, 2005): Pleasence 2007; 0 to 5 month data (Pleasence & Balmer, 2007): Proudfoot 1997; 4 month (Proudfoot *et al.* 1997); Spera, 1994; (Spera *et al.* 1994).

a Risk difference calculated using methods described in Deeks & Higgins (2010) Statistical algorithms in Review Manager 5.2 (Deeks & Higgins, 2010).

b Mean difference calculated using (Salanti, 2013). Statistical algorithms for the Calculator in Review Manager 5.2.

c Employed >20 h per week and considered to be ‘working enough’.

d Employment in JOBS I was defined as working 20 h per week or more PLUS working as many hours as they needed.

e Multivariate analysis of variance (MANOVA).

f Some employment related activity, i.e. temporary, part-time, unpaid, paid, casual or full-time employment or enrolment in part or full-time study.

g Although the Spera *et al.* (1994) study had three arms (*n* = 63) only two of the arms (Writing and No writing *n* = 41) were allocated at random. While we are reporting only from these two study arms for the ANOVA analyses include all three arms. For this analysis only data from 40 participants were available.

h ANOVA across all three intervention arms shows there are significant variation in number employed (*F*_2,59_ = 3.72 *p* = 0.03).

i ORM Ordinal regression model was fitted across the five categories (much better, better, the same, worse, and much worse) and there were no differences between categories by intervention type. When the data were looked at for each category, only one was significant, the people who had the intervention were more likely to score their perception of debt as ‘better’ Wald text χ^2^ = 5.92, *p* = 0.015.

JOBS II evaluated a modified version of JOBS I and stratified participants at baseline according to their risk of depression (high or low). The authors reported a small improvement of depressive symptoms at 2 years for those who received the intervention (standardized linear regression coefficient −0.06, *p* < 0.05) (see Table 2). As in JOBS I, stronger effects of approximately 0.2 s.d. improvements were seen in participants at high risk of depression (around 40% of the trial participants) (interaction: *F*_1,1331_ = 4.10, *p* = 0.043). There was no effect on the Composite Index of Depression Inventory (CIDI), a 9-point scale of likelihood of major depressive episode (MDE) (linear regression: −0.04 s.d. *p* > 0.05). However, using the more stringent criteria of probable (90% likely) to have a MDE (scores of 7 or 8 on CIDI) fewer people in the intervention group had probable MDE at 2 years compared to those in the control (odds ratio 0.61, *p* < 0.05). This intervention was also associated with improved employment at this time point (Vinokur *et al.* 1995*b*,  1996, 2000; Vinokur & Schul, 1997). In the Finnish version of JOBS II, Työhön (‘Let's get to work’), there were improved psychological symptoms (GHQ-12) at 6 months and improved depression symptoms at 6 months and 24 months, although, as described above for JOBS II, the actual size of the reduction in depressive symptoms was small. No analyses stratified by baseline depression risk were reported. In the Työhön study there was no effect on employment at 6 or 24 months (see Tables 2 and 3) (Vuori *et al.* 2002; Vuori & Silvonen, 2005; Vuori & Vinokur, 2005). Rife and colleagues assessed the effects of a similar intervention, more focussed on practical skills training, and reported that the people in the intervention group showed an improvement in depressive symptoms on the Geriatric Depression Scale at 3 months (*p* < 0.05) and more of this group were employed compared to the control group (65% *v.* 26% *p* < 0.01) (see Tables 2 and 3) (Rife, 1992; Rife & Belcher, 1994). Gustafson's small (*n* = 16) trial found no effect of the ‘job club’ intervention on anxiety scores or employment (see Table 2), but was under-powered (Gustafson, 1995). The trials and subgroups where the greatest improvements were seen in employment were those in which participants experienced the greatest improvement in depression (Rife, 1992; Rife & Belcher, 1994; Vinokur *et al.* 1995*b*).

### CBT interventions

Two studies assessed the effect of group CBT on long-term unemployed individuals. The intervention content was similar in both trials, including cognitive restructuring, behaviour modification and homework assignments. However, Proudfoot *et al*. (1997) provided sessions of 3 h per week for 7 weeks (21 h) compared to Harris *et al*. (2002) who provided 11 h over 2 days (see Table 1 and Supplementary Appendix 3). Both studies used an active comparator arm [social support programme that included homework (Proudfoot *et al.* 1997) and a 2-day senior certificate in first aid (Harris *et al.* 2002)] to mimic the attention provided to participants in the intervention arm. The populations were quite different; Proudfoot *et al.* (*n* = 289) enrolled long-term (>12 months) unemployed professionals who were mostly (83%) male, whereas Harris *et al.* (*n* = 195) targeted long-term unemployed individuals in disadvantaged areas who were of lower socioeconomic status and were 55% male (see Table 1) (Harris *et al.* 2002; Proudfoot *et al.* 1997).

Proudfoot *et al.* reported that CBT improved mental health (GHQ-30) scores (difference in means: -1.44, 95% CI −3.20 to 0.32, *p* < 0.05) at 7 weeks but found no effect on the proportion of participants meeting thresholds for a psychiatric ‘case’ (defined as a score >5 on GHQ-30) in the intervention group (21%) compared to the control group (23%) (*p* = 0.78; see Table 2). However, people receiving CBT were more likely to be employed at 7 weeks compared to those in the control group (34% *v.* 13%, *p* = 0.0006; see Table 3) (Proudfoot *et al.* 1997). The second, smaller CBT trial of Harris *et al.* (2002) showed no effect on employment or the mental health indicators (Harris *et al.* 2002); if anything there was evidence of an adverse effect on measures of hopelessness (difference in means: −2.04, 95% CI 0.66 to 3.42) and optimism (difference in means: −2.6, 95% CI −4.10 to 1.10; see Table 2), although there were baseline differences between the study arms with higher levels of self-esteem (Table 2) and shorter durations of unemployment (Table 3) in the control compared to the intervention arm (Harris *et al.* 2002).

### Other interventions

Four other interventions have been evaluated in single trials of 41–402 participants. Telephone debt advice to  people (*n* = 402) who were in debt (recruited from unemployment offices) had no effect on measures of anxiety (Table 2) or on numerous measures of indebtedness (see Table 3) (Pleasence & Balmer, 2007), but only 31% of participants in the intervention group actually received debt advice and 10% of the control (no intervention) group independently sought debt advice (Pleasence & Balmer, 2007). One study (*n* = 41) provided unemployed people with opportunities for expressive writing (20 min over 5 days where they could disclose effects of unemployment) compared to control writing (Spera *et al.* 1994). Expressive writing had no effect on symptoms of anxiety at 3 months (Table 2) but appeared to improve employment (Table 3) (Spera *et al.* 1994). Joseph and colleagues (*n* = 76) assessed the effects of guided imagery (20 min over six sessions) that allowed participants to visualize their success at finding and obtaining employment and also included relaxation techniques compared to control imagery (see Table 2) (Joseph, 1999; Joseph & Greenberg, 2001). People who received guided imagery were more likely to be in employment at 7 weeks but there was no effect on depression (Tables 2 and 3) (Joseph, 1999; Joseph & Greenberg, 2001). Finally, a trial (*n* = 75) of the provision of group emotional competency training for unemployed people compared to no intervention did not present an analysis of the effects emotional competency training on mental health but calculation of the difference in means and 95% CIs showed no effect on (GHQ-12), nor on symptoms of depression, anxiety or stress (see Table 2) (Hodzic *et al.* 2015*a*).

## Discussion

### Main findings

There is consistent evidence from large RCTs in different settings, including three trials with more than 1000 participants, that intensive 1- to 2-week ‘job club’ interventions for unemployed people reduce the risk of depression. The most clinically relevant effects are seen among participants at increased risk of developing depression (around a quarter of participants); in this group, effect sizes of up to 0.5 s.d. improvements in depression scores were seen. Improvement in depression was seen for up to 2 years, although effects on employment were mixed. Larger effects on depression were seen in trials/subgroups with the greatest increases in employment.

The only other intervention investigated in more than one trial was group CBT. There was good evidence of a short-term (3 months) effect on depression symptoms and re-employment in the larger trial that delivered CBT over 7 weeks, though no effect on psychiatric ‘caseness’ (Proudfoot *et al.* 1997). The second showed no beneficial effects (Harris *et al.* 2002). Differences between these two trials may reflect differences in participants [professionals (Proudfoot *et al.* 1997) *v.* people from disadvantaged areas (Harris *et al.* 2002)] and timing/intensity of the intervention [21 h provided weekly over 7 weeks (Proudfoot *et al.* 1997) *v.* 11 h provided over 2 days (Harris *et al.* 2002)].

There is limited evidence for other interventions, but these were all evaluated in single trials with small sample sizes (Spera *et al.* 1994; Joseph & Greenberg, 2001; Hodzic *et al.* 2015*a*) or limited uptake of the intervention (Pleasence & Balmer, 2007).

Risk of bias was a problem for many of the studies with a third of the items rendered not assessable because the items were not reported. Taking into account the high risk of bias of the studies we have to interpret the strength of the evidence with some caution. Studies varied in the quality of reporting important details of interventions. In four a manual was used (Caplan *et al.* 1989; Vinokur *et al.* 1995*b*; Harris *et al.* 2002; Vuori *et al.* 2002) and three included an assessment of fidelity to treatment (Caplan *et al.* 1989; Vinokur *et al.* 1995*b*; Vuori *et al.* 2002). Detailed descriptions of interventions and assessments made to assess fidelity to treatment should be described in future studies (Craig *et al.* 2008).

Evidence of improvement in depressive symptoms in intervention group participants coincided in some studies with higher levels of re-employment (JOBS II high-risk group and Proudfoot *et al.*) (Vinokur *et al.* 1995*b*; Proudfoot *et al.* 1997, 1999; Vinokur *et al.* 2000); however, in some studies this was not the case (JOBS I and Työhön) (Vinokur *et al.* 1991*b*; Price *et al.* 1992; Vuori *et al.* 2002; Vuori & Silvonen, 2005). Some studies looked for aspects of the intervention that might contribute to the change in outcome (mediating effects). There was some evidence in JOBS II that reduced depressive symptoms were associated with reduction of financial strain and reemployment (Vinokur & Schul, 1997). Job search preparedness (self-efficacy and inoculation against setbacks) reduced depressive symptoms and improved employment in JOBS I and Työhön but the authors do not present an analysis on effects of employment on mental health for JOBS I (Vanryn & Vinokur, 1992; Vuori & Vinokur, 2005). Työhön authors did identify a link between reduced financial strain and reduction in depressive symptoms (Vuori & Vinokur, 2005).

### Strengths and limitations

To the best of our knowledge this is the first comprehensive review of RCT evidence of interventions targeted at alleviating the impact on mental health of unemployment and debt in general population samples. Two reviews with a narrower focus solely on ‘job search’ interventions, which included evidence from non-randomized studies, reported that ‘job search’ interventions reduced depression and anxiety and improved employment (Audhoe *et al.* 2010; Liu *et al.* 2014). A strong evidence base in this area is important to inform policy responses to future recessions as these are associated with rises in unemployment, debt, depression, and suicidal behaviour. Our review facilitates an overview of the types of interventions considered to date, providing pointers to what works best to inform future research in this field.

The main limitation is the relatively sparse literature in this field. The only intervention evaluated in large trials in different settings was the ‘job club’ intervention and results for these trials are not presented in such a way as to enable meta-analysis. Thus identifying what works for whom and when was not possible from this data set. There is some evidence from a systematic review that included non-randomized studies that job-search interventions only appeared to improve employment if they included components that developed skills and enhanced motivation, although mediating effects on mental health were not investigated (Liu *et al.* 2014). In JOBS II there was some evidence that the intervention had the greatest effects on mental health among those individuals with low levels of mastery (a composite measure based on self-esteem, self-efficacy and locus of control measures) at baseline, but such effects have not been investigated systematically (Vinokur *et al.* 2000). Suicide (incidence 11.4/1 00 000) and attempted suicide (4/1000), possibly the most severe effect of economic hardship, are thankfully relatively rare (WHO, 2014, 2016); reported trials are underpowered to detect any effect on these indicators and were not evaluated in the studies we reviewed. Our focus was on trials evaluating mental health, the key risk factor for suicide and we assumed that interventions having an impact on mental health will in turn influence suicide attempts and suicide. The studies we identified used a range of outcome measures (GHQ-12, GHQ-30, SF-36, GDS, PSSS, Beck Hopelessness scale, POMS, CIDI, CES-D HSCL-90, STAI) designed to measure different aspects of mental health and ranging from disorder specific scales (e.g. CES-D) to screens for common mental disorder (GHQ-12). We excluded 20 reports of RCTs because they did not report any measures for mental health and we would recommend that any future trialists investigating interventions for debt and unemployment collect and publish such outcomes.

*A priori* we decided not to review the literature on specific groups, e.g. people with severe mental illness and single mothers, although some of the included trials did focus on population subgroups [e.g. unemployed professionals (Proudfoot *et al.* 1997), long-term unemployed living in deprived areas (Harris *et al.* 2002)]. Literature in this area is rather old and largely focused on the unemployed/job loss. The one trial of debt advice had to be stopped early because of high levels of loss to follow-up (Pleasence & Balmer, 2007; Pleasence, 2008).

### Findings in the context of the wider literature

The focus of our review has been on the relatively few RCTs in this field. Conducting trials in the context of economic recession is challenging as the rapid rises in job loss and debt require timely policy responses. Timescales for obtaining research funding to conduct RCTs, and time delays in obtaining research ethics approval (Salman *et al.* 2014), means that by the time a trial is funded the most acute period of economic difficulty may have passed. Three previous systematic reviews have all remarked upon the absence of evidence of health benefits for public health interventions for people on low income. One found 10 RCTs on income supplementation by searching medical and sociological literature but none had measured any aspect of health (Connor *et al.* 1999). A broader search including grey literature for welfare advice delivered in a healthcare setting found 55 studies (one RCT) and concluded that income was improved but could not comment on health (Adams *et al.* 2006). A third searched medical literature for vocational interventions for unemployed and found weak evidence of no effect on mental health (Audhoe *et al.* 2010).

Several observational studies have investigated the impact of different policy responses to periods of recession and provide additional pointers to effective interventions. Stuckler *et al.*’s analysis of the association of changes in unemployment with changes in suicide mortality in 26 European Union countries between 1970 and 2007 indicates that government spending on active labour market programmes mitigated the effect rises in unemployment on suicide (Stuckler *et al.* 2009). Components of active labour market programmes include classroom or on the job training, job search assistance or sanctions for failing to search, subsidized private sector employment and subsidized public sector employment. Notably, some elements of these programmes were included in the ‘job club’ (interventions described above) (Caplan *et al.* 1989; Vinokur *et al.* 1991*a*, *b*, 1995*a*; Price *et al.* 1992; Vanryn & Vinokur, 1992).

Two recent ecological studies conducted during the 2008 recession provide evidence of the beneficial effects of providing generous welfare benefits to people who are out of work. An analysis of US state-level suicide data showed that states providing more generous unemployment benefits experienced lower recession-related rises in suicide than those providing less generous welfare support (Cylus *et al.* 2014). In an ecological analysis of data from 30 countries, there was a graded association between a country's spending on unemployment protection and the effect of unemployment rises on suicide (Norström & Grönqvist, 2014).

### Future research agenda

Whilst some authors did report their RCTs with reference to CONSORT guidelines (Pleasence & Balmer, 2007; Schultz *et al.* 2010) we would urge authors of future studies to follow this lead to allow accurate assessments of risk-of bias and clarity of analysis and treatment effects (Turner *et al.* 2012). Some trials had low recruitment rates and, given the likely financial difficulties faced by research participants in this field, trialists could attempt to increase participation by providing incentives or compensation for participants’ time, as five of the 11 studies in this review did (see Supplementary Appendix 3).

Recent research into suicides occurring during a period of recession indicated that those whose suicide appeared to be related to consequences of recession were largely still in work, cohabiting, with financial dependents but had no contact with secondary-care psychiatric and little recent contact with primary-care services (Coope *et al.* 2015). This indicates the need for research into how best to (*a*) identify those at risk of adverse mental health outcomes during recession, and (*b*) intervene to reduce risk among those not in contact with services. In the UK's recent recession, policy responses included training front-line job centre and debt collection staff (Fitch & Davey, 2010; Domokos, 2011). Evaluation of these and similar interventions would inform responses to future recessions. From a healthcare perspective, pragmatic trials in primary care such as the DeCoDer trial will provide useful additional evidence for primary care type interventions to address economic hardship and depression and anxiety (DECODER, 2014). In addition the impact of training primary-care and specialist mental health staff in appraising financial difficulties and signposting them to appropriate statutory and voluntary sector organizations may be worthwhile and form the basis of future evaluative research (Adams *et al.* 2006; Harris & Harris, 2009; Barnes *et al.* 2016). Future RCTs could usefully include measures to help identify what intervention works for whom and why. They might specifically build on work by studies in this review and measure mediating effects e.g. self-efficacy, debt management skills etc. Stratification to create *a priori* subgroups of participants would help identify which groups were benefitting or not from the intervention. Other potential subgroups could include age, socioeconomic position, or gender. Nested qualitative research could be used to identify potential barriers and facilitators to intervention uptake and adherence among participants and be useful in helping to identify time critical aspects of the intervention, e.g. were participants successful at gaining employment, managing debt, etc. because their mood lifted or vice versa? Researchers could try to publish across both socio-legal and medical disciplines and future systematic reviews should search both literature sources.

## Conclusions

There is reasonably consistent evidence from large RCTs that short, 1- to 2-week ‘job club’ interventions can reduce depressive symptoms in high-risk, unemployed people up to 2 years. Evidence for CBT is mixed and for other interventions it is limited. Further high-quality RCTs are urgently needed. Such trials might usefully focus on interventions to help individuals with financial difficulties and debt, as most of the literature to date as focussed on provision of help to the unemployed, although the two issues are closely inter-related.

## Supplementary material

The supplementary material for this article can be found at https://doi.org/10.1017/S0033291716002944.

## References

[ref1] Adams J, White M, Moffatt S, Howel D, Mackintosh J (2006). A systematic review of the health, social and financial impacts of welfare rights advice delivered in healthcare settings. BMC Public Health 6, 28. 16571122 10.1186/1471-2458-6-81 PMC1440855

[ref2] Audhoe SS, Hoving JL, Sluiter JK, Frings-Dresen MH (2010). Vocational interventions for unemployed: effects on work participation and mental distress. A systematic review. Journal of Occupational Rehabilitation 20, 1–13. 20039106 10.1007/s10926-009-9223-y

[ref3] Azrin N (1978). The job-finding club as a method for obtaining employment for welfare-eligible clients: Demonstration, evaluation and counselor training. US Department of labour Report No DLMA-5t-17-76-04.

[ref4] Azrin NH, Flores T, Kaplan SJ (1975). Job-finding club: a group-assisted program for obtaining employment. Behaviour Research and Therapy 13, 17–27.

[ref5] Azrin NH, Philip RA (1979). The job club method for the job handicapped: a comparative outcome study. Rehabilitation Counseling Bulletin 23, 144–155.

[ref6] Baicker K, Taubman SL, Allen HL, Bernstein M, Gruber JH, Newhouse JP, Schneider EC, Wright BJ, Zaslavsky AM, Finkelstein AN, Oregon Health Study G, Carlson M, Edlund T, Gallia C, Smith J (2013). The Oregon experiment-effects of medicaid on clinical outcomes. New England Journal of Medicine 368, 1713–1722. 23635051 10.1056/NEJMsa1212321 PMC3701298

[ref7] Barnes MC, Gunnell D, Davies R, Hawton K, Kapur N, Potokar J, Donovan JL (2016). Understanding vulnerability to self-harm in times of economic hardship and austerity: a qualitative study. BMJ Open bmjopen-2015-010131. 10.1136/bmjopen-2015-010131 PMC4762121 26888729

[ref8] Barr B, Taylor-Robinson D, Stuckler D, Loopstra R, Reeves A, Wickham S, Whitehead M (2015). Fit-for-work or fit-for-unemployment? Does the reassessment of disability benefit claimants using a tougher work capability assessment help people into work? Journal of Epidemiology and Community Health jech-2015–206333. 10.1136/jech-2015-206333 26646692

[ref9] Burke-Miller J, Razzano LA, Grey DD, Blyler CR, Cook JA (2012). Supported employment outcomes for transition age youth and young adults. Psychiatric Rehabilitation Journal 35, 171–179. 22246115 10.2975/35.3.2012.171.179

[ref10] Caplan RD, Vinokur AD, Price RH, van Ryn M (1989). Job seeking, reemployment, and mental health: a randomized field experiment in coping with job loss. Journal of Applied Psychology 74, 759–769. 2793774 10.1037/0021-9010.74.5.759

[ref11] Chang S-S, Stuckler D, Yip P, Gunnell D (2013). Impact of 2008 global economic crisis on suicide: time trend study in 54 countries. British Medical Journal BMJ 2013;347:f5239. 10.1136/bmj.f5239 PMC3776046 24046155

[ref12] Connor J, Rodgers A, Priest P (1999). Randomised studies of income supplementation: a lost opportunity to assess health outcomes. Journal of Epidemiology & Community Health 53, 725–730. 10656103 10.1136/jech.53.11.725 PMC1756807

[ref13] Coope C, Donovan J, Wilson C, Barnes M, Metcalfe C, Hollingworth W, Kapur N, Hawton K, Gunnell D (2015). Characteristics of people dying by suicide after job loss, financial difficulties and other economic stressors during a period of recession (2010–2011): a review of coroners’ records. Journal of Affective Disorders 183, 98–105. 26001669 10.1016/j.jad.2015.04.045

[ref14] Corcoran P, Griffin E, Arensman E, Fitzgerald AP, Perry IJ (2015). Impact of the economic recession and subsequent austerity on suicide and self-harm in Ireland: an interrupted time series analysis. International Journal of Epidemiology 10.1093/ije/dyv058. pp. 969–977. 26082406 10.1093/ije/dyv058

[ref15] Craig P, Dieppe P, Macintyre S, Michie S, Nazareth I, Petticrew M (2008). Developing and evaluating complex interventions: the new Medical Research Council guidance. British Medical Journal BMJ 2008;337:a1655. 10.1136/bmj.a1655 PMC2769032 18824488

[ref16] Cylus J, Glymour MM, Avendano M (2014). Do generous unemployment benefit programs reduce suicide rates? A state fixed-effect analysis covering 1968–2008. American Journal of Epidemiology 180, 45–52. 24939978 10.1093/aje/kwu106 PMC4070935

[ref17] DECODER (2014). DeCoDer trial debt counselling for depression in primary care: an adaptive randomised controlled trial (Project record). *Health Technology Assessment Database* 3 HTA-32014000443. 10.3310/hta21350 PMC5502372 28648148

[ref18] Deeks J, Higgins DJ on behalf of the Cochrane Statistical Methods Group (2010). Statistical algorithms in Review Manager 5.2. The Cochrane Collaboration. pp 1–11. Retrieved from http://ims.cochrane.org/revman/documentation/Statistical-methods-inRevMan-5.pdf

[ref19] Derogatis LR, Lipman RS, Rickels K, Uhlenhuth EH, Covi L (1974). The Hopkins Symptom Checklist (HSCL): a self-report symptom inventory. Behavioral Science 19, 1–15. 4808738 10.1002/bs.3830190102

[ref20] Domokos J (2011). Jobcentre staff ‘sent guidelines on how to deal with claimants’ suicide threats’ In The Guardian. The Guardian: UK (https://www.theguardian.com/society/2011/may/08/jobcentre-staff-guidelines-suicide-threats).

[ref21] Fernald LC, Hamad R, Karlan D, Ozer EJ, Zinman J (2008). Small individual loans and mental health: a randomized controlled trial among South African adults. BMC Public Health 8, 409. 19087316 10.1186/1471-2458-8-409 PMC2647927

[ref22] Finkelstein A, Taubman S, Wright B, Bernstein M, Gruber J, Newhouse JP, Allen H, Baicker K, Or, Oregon Health Study Group (2012). The Oregon health insurance experiment: evidence from the first year. Quarterly Journal of Economics 127, 1057–1106. 23293397 10.1093/qje/qjs020 PMC3535298

[ref23] Fitch C, Davey R (2010). Debt collection and Mental Health: Ten Steps to Improve Recovery. Royal College of Psychiatrists and the Money Advice Trust: London, p. 20.

[ref24] Fitch C, Hamilton S, Bassett P, Davey R (2011). The relationship between personal debt and mental health: a systematic review. Mental Health Review Journal 16, 153–166.

[ref25] Forgatch MS, DeGarmo DS (2007). Accelerating recovery from poverty: prevention effects for recently separated mothers. Journal of Early and Intensive Behavior Intervention 4, 681–702. 19043620 10.1037/h0100400 PMC2587348

[ref26] Goldberg DP, Gater R, Sartorius N, Ustun TB, Piccinelli M, Gureje O, Rutter C (1997). The validity of two versions of the GHQ in the WHO study of mental illness in general health care. Psychological Medicine 27, 191–197. 9122299 10.1017/s0033291796004242

[ref27] Gray D (1983). A job club for older job seekers: an experimental evaluation. Journal of Gerontology 38, 363–368. 6841934 10.1093/geronj/38.3.363

[ref28] Gustafson DJ (1995). Job seeking, reemployment, and mental health: An intervention experiment in coping with job loss. Ph.D. Thesis. California State University: Long Beach.

[ref29] Harris E, Harris MF (2009). Reducing the impact of unemployment on health: revisiting the agenda for primary health care. Medical Journal of Australia 191, 119–122. 19619102 10.5694/j.1326-5377.2009.tb02709.x

[ref30] Harris E, Lum J, Rose V, Morrow M, Comino E, Harris M (2002). Are CBT interventions effective with disadvantaged job-seekers who are long-term unemployed? Psychology, Health and Medicine 7, 401–410.

[ref31] Haw C, Hawton K, Gunnell D, Platt S (2015). Economic recession and suicidal behaviour: possible mechanisms and ameliorating factors. International Journal of Social Psychiatry 61, 73–81. 24903684 10.1177/0020764014536545

[ref32] Higgins J, Altman D, Sterne JAC (2011). Chapter 8: assessing risk of bias in included studies In Cochrane Handbook for Systematic Reviews of interventions Version 5.1 [updated March 2011] (ed. Higgins JPT and Green S). The Cochrane Collaboration The Cochrane Library (www.cochrane-handbook.org).

[ref33] Higgins J, Green S (2011). Cochrane Handbook for Systematic Reviews of interventions Version 5.1 [updated March 2011]. The Cochrane Library, The Cochrane Collaboration (2011). (www.cochrane-handbook.org). The Cochrane Collaboration The Cochrane Library.

[ref34] Hodzic S, Ripoll P, Bernal C, Zenasni F (2015 *a*). The effects of emotional competences training among unemployed adults: a longitudinal study. Applied Psychology. Health and Well-being 7, 275–292. 26173636 10.1111/aphw.12048

[ref35] Hodzic S, Ripoll P, Lira E, Zenasni F (2015 *b*). Can intervention in emotional competences increase employability prospects of unemployed adults? Journal of Vocational Behavior 88, 28–37.

[ref36] Jagannathan R, Camasso MJ, Sambamoorthi U (2010). Experimental evidence of welfare reform impact on clinical anxiety and depression levels among poor women. Social Science & Medicine 71, 152–160. 20434251 10.1016/j.socscimed.2010.02.044

[ref37] Joseph LM (1999). The effects of guided mental imagery on subsequent reemployment success in recently laid-off white-collar workers. Dissertation Abstracts International: Section B: The Sciences and Engineering 60, 1337.

[ref38] Joseph LM, Greenberg MA (2001). The effects of a career transition program on reemployment success in laid-off professionals. Consulting Psychology Journal: Practice & Research Summer 53, 169–181.

[ref39] Katikireddi SV, Niedzwiedz CL, Popham F (2012). Trends in population mental health before and after the 2008 recession: a repeat cross-sectional analysis of the 1991–2010 Health Surveys of England. BMJ Open 2 bmjopen-2012–001790. 10.1136/bmjopen-2012-001790 PMC3488736 23075569

[ref40] Kneipp SM, Kairalla JA, Lutz BJ, Pereira D, Hall AG, Flocks J, Beeber L, Schwartz T (2011). Public health nursing case management for women receiving temporary assistance for needy families: a randomized controlled trial using community-based participatory research. American Journal of Public Health 101, 1759–1768. 21778474 10.2105/AJPH.2011.300210 PMC3154225

[ref41] Kotsou I, Nelis D, Gregoire J, Mikolajczak M (2011). Emotional plasticity: conditions and effects of improving emotional competence in adulthood. Journal of Applied Psychology 96, 827–839. 21443316 10.1037/a0023047

[ref41a] Kukla M, Bond GR (2009). The working alliance and employment outcomes for people with severe mental illness enrolled in vocational programs. Rehabilitation Psychology 54, 156–163. 10.1037/a0015596 19469605

[ref42] Lefebvre C, Mannheimer E, Glanville J, On Behalf of the Cochrane Retrieval Methods Group (2011). Chapter 6: searching for studies In Cochrane Handbook for Systematic Reviews of Interventions Version 5.1 [updated March 2011]. (ed. Higgins JPT, Green S). The Cochrane Collaboration The Cochrane Library (www.cochrane-handbook.org).

[ref43] Liberati A, Altman DG, Tetzlaff J, Mulrow C, Gøtzsche PC, Ioannidis JPA, Clarke M, Devereaux PJ, Kleijnen J, Moher D (2009). The PRISMA statement for reporting systematic reviews and meta-analyses of studies that evaluate healthcare interventions: explanation and elaboration. British Medical Journal 339 http://www.bmj.com/content/339/bmj.b2700 10.1136/bmj.b2700 PMC2714672 19622552

[ref44] Liu S, Huang JL, Wang M (2014). Effectiveness of job search interventions: a meta-analytic review. Psychological Bulletin July 140, 1009–1041. 10.1037/a0035923 24588365

[ref45] Moore T, Gunnell D, Metcalfe C, Kapur N, Hawton K (2015). Effects of interventions to ameliorate the impact of unemployment and economic hardship on mental health in the general population. *PROSPERO Database*. CRD42015019822. 10.1017/S0033291716002944 PMC5426338 27974062

[ref46] Morris PA, Hendra R (2009). Losing the safety net: how a time-limited welfare policy affects families at risk of reaching time limits. Developmental Psychology 45, 383–400. 19271826 10.1037/a0014960 PMC3208319

[ref47] Nieuwenhuijsen K, Faber B, Verbeek JH, Neumeyer-Gromen A, Hees HL, Verhoeven AC, van der Feltz-Cornelis CM, Bültmann U (2014). Interventions to improve return to work in depressed people Cochrane Database of Systematic Reviews 2014, Issue 12. CD006237. DOI: 10.1002/14651858.CD006237.pub3.. 10.1002/14651858.CD006237.pub3 25470301

[ref48] Norström T, Grönqvist H (2014). The great Recession, unemployment and suicide. Journal of Epidemiology and Community Health 69, 110–116. 25339416 10.1136/jech-2014-204602 PMC4316842

[ref49] Pleasence P (2008). Trials and tribulations: conducting randomized experiments in a socio-legal setting. Journal of Law and Society 35, 8–29.

[ref50] Pleasence P, Balmer NJ (2007). Changing fortunes: results from a randomized trial of the offer of debt advice in England and wales. Journal of Empirical Legal Studies 4, 651–673.

[ref51] Price RH, Van Ryn M, Vinokur AD (1992). Impact of a preventive job search intervention on the likelihood of depression among the unemployed. Journal of Health & Social Behavior 33, 158–167. 1619263

[ref52] Proudfoot J, Gray J, Carson J, Guest D, Dunn G (1999). Psychological training improves mental health and job-finding among unemployed people. International Archives of Occupational and Environmental Health 72, S40–S42. 10197475

[ref53] Proudfoot J, Guest D, Carson J, Dunn G, Gray J (1997). Effect of cognitive-behavioural training on job-finding among long-term unemployed people. Lancet 350, 96–100. 9228961 10.1016/S0140-6736(96)09097-6

[ref54] Rife J (1992). Reducing depression and increasing job placement success of older workers. Clinical Gerontologist 12, 81–85.

[ref55] Rife JC, Belcher JR (1994). Assisting unemployed older workers to become reemployed: an experimental evaluation. Research on Social Work Practice 4, 3–13.

[ref56] Salanti G on behalf of the Cochrane Statistical Methods Group. (2013). Statistical algorithms for the Calculator in Review Manager 5.2. The Cochrane Collaboration. RevMan avialable from http://tech.cochrane.org/revman/download

[ref57] Salman RA-S, Beller E, Kagan J, Hemminki E, Phillips RS, Savulescu J, Macleod M, Wisely J, Chalmers I (2014). Increasing value and reducing waste in biomedical research regulation and management. Lancet 383, 176–185. 24411646 10.1016/S0140-6736(13)62297-7 PMC3952153

[ref58] Salokangas R, Stengard E, Poutanen O (1994). uusi väline depression torjuntaan [a new screening test for depressive symptoms]. Duodecim 110, 1141–1148. 7497919

[ref59] Schulz KF, Altman DG, Moher D (2010). CONSORT 2010 Statement: updated guidelines for reporting parallel group randomized trials. Annals of Internal Medicine 152, 726–732. 20335313 10.7326/0003-4819-152-11-201006010-00232

[ref60] Spera SP, Buhrfeind ED, Pennebaker JW (1994). Expressive writing and coping with job loss. Academy of Management Journal 37, 722–733.

[ref61] Stuckler D, Basu S, Suhrcke M, Coutts A, McKee M (2009). The public health effect of economic crises and alternative policy responses in Europe: an empirical analysis. Lancet 374, 315–323. 19589588 10.1016/S0140-6736(09)61124-7

[ref62] Tsang HW, Fung KM, Leung AY, Li SM, Cheung WM (2010). Three year follow-up study of an integrated supported employment for individuals with severe mental illness. Australian & New Zealand Journal of Psychiatry 44, 49–58. 20073567 10.3109/00048670903393613

[ref63] Turner L, Shamseer L, Altman DG, Weeks L, Peters J, Kober T, Dias S, Schulz KF, Plint AC, Moher D (2012). Consolidated standards of reporting trials (CONSORT) and the completeness of reporting of randomised controlled trials (RCTs) published in medical journals. Cochrane Database of Systematic Reviews 11. doi:10.1002/14651858.MR000030.pub2 PMC7386818 23152285

[ref64] Vanryn M, Vinokur AD (1992). How did it work - An examination of the mechanisms through which an intervention for the unemployed promoted job-search behavior. American Journal of Community Psychology 20, 577–597. 1485612 10.1007/BF00941773

[ref65] van Stolk C, Hofman J, Hafner M, Janta B (2014). Psychological Wellbeing and Work: Improving Service Provision and Outcomes. Department for Work and Pensions and Department of Health: London, UK. 10.7249/rhq-04-01-09 PMC5051977 28083323

[ref66] Vinokur AD, Price RH, Caplan RD (1991 *a*). From field experiments to program implementation: assessing the potential outcomes of an experimental intervention program for unemployed persons. American Journal of Community Psychology 71, 166–179. 10.1007/BF00937991 1755435

[ref67] Vinokur AD, Price RH, Caplan RD (1996). Hard times and hurtful partners: how financial strain affects depression and relationship satisfaction of unemployed persons and their spouses. Journal of Personality & Social Psychology 71, 166–179. 8708998 10.1037//0022-3514.71.1.166

[ref68] Vinokur AD, Price RH, Caplan RD, van Ryn M, Curran J (1995 *a*). The Jobs I preventive intervention for unemployed individuals: short- and long-term effects on reemployment and mental health In Job Stress Interventions, pp. 125–138. American Psychological Association: Washington, DC, USA.

[ref69] Vinokur AD, Price RH, Schul Y (1995 *b*). Impact of the JOBS intervention on unemployed workers varying in risk for depression. American Journal of Community Psychology 23, 39–74. 7572826 10.1007/BF02506922

[ref70] Vinokur AD, Schul Y (1997). Mastery and inoculation against setbacks as active ingredients in the JOBS intervention for the unemployed. Journal of Consulting & Clinical Psychology 65, 867–877. 9337505 10.1037//0022-006x.65.5.867

[ref71] Vinokur AD, Schul Y, Vuori J, Price RH (2000). Two years after a job loss: long-term impact of the JOBS program on reemployment and mental health. Journal of Occupational Health Psychology 5, 32–47. 10658883 10.1037//1076-8998.5.1.32

[ref72] Vinokur AD, van Ryn M, Gramlich EM, Price RH (1991 *b*). Long-term follow-up and benefit-cost analysis of the Jobs Program: a preventive intervention for the unemployed. Journal of Applied Psychology 76, 213–219. 1905293 10.1037/0021-9010.76.2.213

[ref73] Vuori J, Silvonen J (2005). The benefits of a preventive job search program on re-employment and mental health at 2-year follow-up. Journal of Occupational and Organizational Psychology 78, 43–52.

[ref74] Vuori J, Silvonen J, Vinokur AD, Price RH (2002). The Tyohon job search program in Finland: benefits for the unemployed with risk of depression or discouragement. Journal of Occupational Health Psychology 7, 5–19. 11827233 10.1037//1076-8998.7.1.5

[ref75] Vuori J, Vinokur AD (2005). Job-search preparedness as a mediator of the effects of the tyohon job search Intervention on re-employment and mental health. Journal of Organizational Behavior 26, 275–291.

[ref76] Ware J Jr., Kosinski M, Keller S (1994). SF 36 Physical and Mental Health Summary Scales: A User's Manual. The Health Institute, New England Medical Center.

[ref77] Watson D, Pennebaker J (1989). Health complaints, stress and distress. Psychological Review 96, 234–254. 2710874 10.1037/0033-295x.96.2.234

[ref78] WHO (2011). Impact of Economic Crises on Mental Health. Copenhagen, Denmark. p. 34.

[ref79] WHO (2014). Preventing Suicide: A Global Imperative. WHO: Geneva, Switzerland p. 92 (http://www.who.int/mental_health/suicide-prevention/world_report_2014/en/)

[ref80] WHO (2016). Age-Standardized Suicide Rates (per 100 000 population). WHO: Geneva, Switzerland (http://www.who.int/gho/mental_health/suicide_rates/en/).

[ref81] Wiggins M, Oakley A, Roberts I, Turner H, Rajan L, Austerberry H, Mujica R, Mugford M (2004). The social support and family health study: a randomised controlled trial and economic evaluation of two alternative forms of postnatal support for mothers living in disadvantaged inner-city areas. Health technology assessment *(*Winchester, England*)* 8, iii, ix-x, 1–120. 10.3310/hta8320 15298823

